# Pancreatic Islet Purification from Large Mammals and Humans Using a COBE 2991 Cell Processor versus Large Plastic Bottles

**DOI:** 10.3390/jcm10010010

**Published:** 2020-12-23

**Authors:** Hirofumi Noguchi

**Affiliations:** Department of Regenerative Medicine, Graduate School of Medicine, University of the Ryukyus, Okinawa 903-0215, Japan; noguchih@med.u-ryukyu.ac.jp; Tel.: +81-98-895-1696; Fax: +81-98-895-1684

**Keywords:** islet transplantation, islet purification, COBE 2991 cell processor, large cylindrical plastic bottles, continuous density gradient

## Abstract

The islet purification step in clinical islet isolation is important for minimizing the risks associated with intraportal infusion. Continuous density gradient with a COBE 2991 cell processor is commonly used for clinical islet purification. However, the high shear force involved in the purification method using the COBE 2991 cell processor causes mechanical damage to the islets. We and other groups have shown human/porcine islet purification using large cylindrical plastic bottles. Shear stress can be minimized or eliminated using large cylindrical plastic bottles because the bottles do not have a narrow segment and no centrifugation is required during tissue loading and the collection processes of islet purification. This review describes current advances in islet purification from large mammals and humans using a COBE 2991 cell processor versus large cylindrical plastic bottles.

## 1. Introduction

Pancreatic islet transplantation is a minimally invasive treatment option to achieve tight glucose control in patients with type 1 diabetes who experience frequent severe hypoglycemia despite maximal care [1,2,3,4,5]. Although the techniques of islet isolation have been gradually improving [6,7,8,9,10,11], the islet yields using current techniques are usually fewer than half of the islets from a donor pancreas, and barely half of the processed pancreata effectively reach the threshold for clinical transplantation in most centers. One of the most important steps for islet isolation is islet purification. The islets, which represent only 2–5% of the pancreas, are separated by a purification step, resulting in the reduction of the tissue volume necessary for implantation. Therefore, the procedure of islet purification reduces the risks associated with islet infusion through the portal vein such as increased portal pressure and thrombosis, increased digestive enzymes from dying acinar cells, and immunoreaction to dying acinar cells after transplantation in unpurified islet preparations. On the other hand, the number of islets decrease after islet purification. This is due to the presence of islets in less pure fractions after islet purification and/or the death of islets by various stressors such as exposure to cytokines/chemokines and mechanical stress [12,13,14,15].

The standard method of islet purification is centrifugation by density gradients, as the density between exocrine tissue and islets is different. It has been reported that the densities of human islets and exocrine tissue are 1.075 and 1.079 g/cm^3^ in 300 mOsm/kg solution [16] and 1.079 and 1.093 g/cm^3^ in 400 mOsm/kg solution [16,17], respectively. A semiautomated computerized COBE 2991 cell processor is commonly used as is considered the gold standard method [1,2]. We and other groups have reported islet purification from large mammals and humans using large cylindrical plastic bottles [18,19,20,21,22,23,24], which substantially improved the efficacy in comparison to standard purification using a COBE 2991 cell processor [18]. This review describes current advances in islet purification from the pancreas of large mammals and humans using a COBE 2991 cell processor versus large plastic bottles.

## 2. Continuous Density Gradient with a COBE 2991 Cell Processor

Standard human islet purification is performed by a COBE 2991 cell processor (TERUMO BCT, Lakewood, CO, US) [1,2,25,26,27,28]. Lake et al. firstly promoted the COBE 2991 cell processor for large-scale purification of human islets in 1989 [29]. They used discontinouos gradiants of bovine serum albumin for human islet purification. The mean purity for 15 human islet purifications was 65% with high yields, and the viability of islets by the COBE 2991 cell processor was no different in function compared with handpicked islets. Their data suggest that the technique using the COBE 2991 cell processor allows isolation of intact, viable human islets of sufficient purity for human islet transplantation [29].

In discontinuous density gradients, islets/exocrine tissue are separated simply on the basis of whether they float or sink. The islets concentrate in the steep areas of the gradient at each interface. The discontinuous density gradient is a convenient compression procedure for preparative purposes. However, it is also a very dangerous method when the density of islets is higher or the density of exocrine tissue is lower. On the other hand, islets/exocrine tissue are distributed widely in continuous density gradients [30,31].

**Figure 1:**
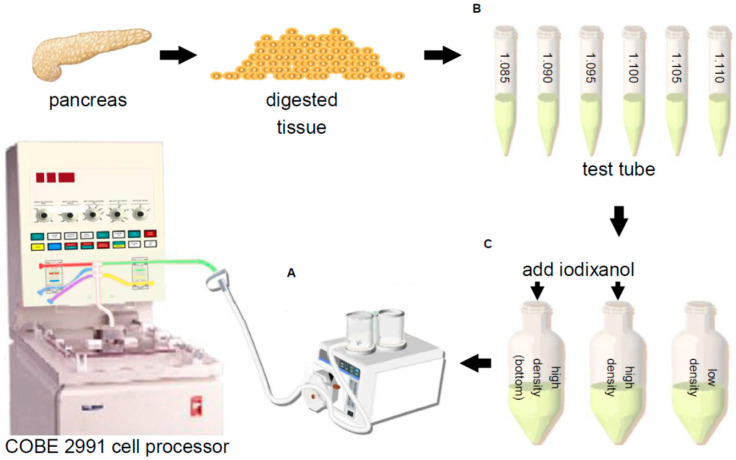
Islet purification using a COBE 2991 cell processor. (**A**) Continuous density gradient. The standard purification procedure for human islet preparations was performed using the COBE 2991 cell processor, gradient maker, and high/low-density solutions. (**B**) Test tubes. Before islet purification, the density of the digested tissue was calculated using six 5 mL test tubes of different densities (i.e., 1.085, 1.090, 1.095, 1.100, 1.105, 1.110). (**C**) Controlled density gradient. According to the outcome of the test tubes, the high density of the purification solutions was controlled (1.085–1.110 g/cm^3^) by changing the volumetric ratio of the iodixanol and purification solutions.

Continuous density gradients in human islet isolation were firstly used as an analytical tool of islet and exocrine densities [30]. Robertson et al. assessed islet yield and purity, following collagenase digestion of the human pancreas by liner mini continuous density gradients using a two-chamber gradient maker [31]. They showed that the percentage of exocrine contamination was 0% to 55% (mean 12%), when the percentage of islet recovery was 60%, suggesting that the methodology is applicable to improving islet purification [31]. They also showed that large-scale continuous density gradient was able to be produced by the COBE 2991 cell processor [32]. After the Edmonton protocol was announced [1], continuous density gradients by the COBE 2991 cell processor were recognized as the gold standard method for human islet purification (Figure 1A). The percentage of islets recovered from continuous density gradients using Ficoll solutions is 55–65% [7,33,34].

Differences in donor characteristics, pancreas procurement, preservation solutions, and islet isolation procedure, itself, affect the density of islets/exocrine tissue [16,35,36,37]. Cold ischemia time and warm ischemia-associated factors during procurement affect cell life/death, thereby influencing islet and acinar cell density and, subsequently, purification. On the other hand, the density of islets/exocrine tissue from small animals is relatively stable because the pancreata from living donors are commonly used and the islet isolation procedure of small animals is simple. Amounts of 1.100 g/cm^3^ of high-density solution and 1.077 g/cm^3^ of low-density solution are commonly used for human islet purification. When the exocrine density is less than 1.100 g/cm^3^, contamination of exocrine tissue in islet fractions often occurs. On the other hand, when there are a lot of embedded islets and the islet density is more than 1.100 g/cm^3^, most of the islets are lost because of sedimentation of the embedded islets in the COBE bag. We measured the densities of digested tissue (>95% exocrine tissue) in 28 human islet isolations [25]. The density of digested tissue in 14.3% of the isolations was 1.085 g/cm^3^, the density in 32.1% was 1.090 g/cm^3^, the density in 46.4% was 1.095 g/cm^3^, the density in 3.6% was 1.100 g/cm^3^, and the density in 3.6% was 1.105 g/cm^3^. These data suggest that the density varies with the individual isolation and that human islet purification is difficult in some cases.

## 3. Purification Solutions

Before the Edmonton protocol was announced [1], bovine serum albumin (BSA) [29,31,32] or Ficoll-containing solutions [26,27,28] were mainly used for islet purification from large mammals and humans. It has been reported that a discontinuous gradient of BSA improves the yield and purity of rat islets compared with Ficoll [38]. The osmolality of BSA remains unchanged with increasing density [38], while Ficoll has been shown to produce a marked non-liner increase in osmolality down the gradient [39,40]. The viscosity of BSA changes very little over the density range required for the study compared with Ficoll [30]. Moreover, it has been reported that exposure to a sucrose-based Ficoll density gradient and endotoxins during isolation may cause release of inflammatory mediators in vitro [28,41,42]. However, Shapiro et al. applied Ficoll solutions to human islet purification in the Edmonton protocol because they avoid the use of xenoprotein products [1].

Iodixanol-containing purification solutions have been recently reported in clinical islet transplantation at a limited number of centers [4,5,6,25,43,44]. Iodixanol is a nonionic, iso-osmolar radiocontrast agent and is used in patients for intravenous administration. Iodixanol is also used as a density gradient medium and has a lower viscosity than Ficoll. Therefore, iodixanol is a safe agent and may be less forceful for islets. It has been reported that the β-cell mass after culture for 48 h significantly improved in the iodixanol group when compared to that in the Ficoll group, and that the production of cytokines, such as interferon-γ (IFN-γ), interleukin-1β (IL-1β), and tumor necrosis factor-α (TNF-α), in the iodixanol group was significantly lower during the 48 h culture after isolation than in the Ficoll group [44]. IFN-γ, IL-1β, and TNF-α are inflammatory cytokines and are well-known to induce islet apoptosis [8,45]. Therefore, the reduction of these cytokines using iodixanol-containing purification solutions may improve islet transplantation outcomes.

## 4. Controlled Density Gradient

The variety of tissue density with the individual isolation has profound implications for the difficulty of performing islet purification. We and some groups have reported that calculation of the tissue density and optimization of density gradients before islet purification improve islet purification recovery [25,46,47]. Our group has used iodixanol and a preservation solution (MK solution; extracellular-type trehalose-containing Kyoto solution with ulinastatin) to generate a new purification solution (IK solution; iodixanol + MK solution). Before islet purification, we calculated the density of the digested tissue using six 5 mL test tubes of different densities (i.e., 1.085, 1.090, 1.095, 1.100, 1.105, and 1.110) (Figure 1B). According to the results of the test tubes, the high density of the purification solutions was controlled (1.085–1.110 g/cm^3^) by changing the volumetric ratio of iodixanol and IK solutions (Figure 1C). Using the controlled density gradient, the post-purification recovery rate was improved (84.9%) compared to the standard continuous gradient (55.6%) [25]. Anazawa et al. showed an analytical test gradient system (ATGS) using a single conical tube with a continuous gradient before the actual COBE purification [6]. Exocrine contamination in allograft preparations and sedimentation of islets in the COBE bag in autograft preparations were reduced by using ATGS and controlled density gradient.

## 5. Islet Purification Using Large Bottles

**Figure 2:**
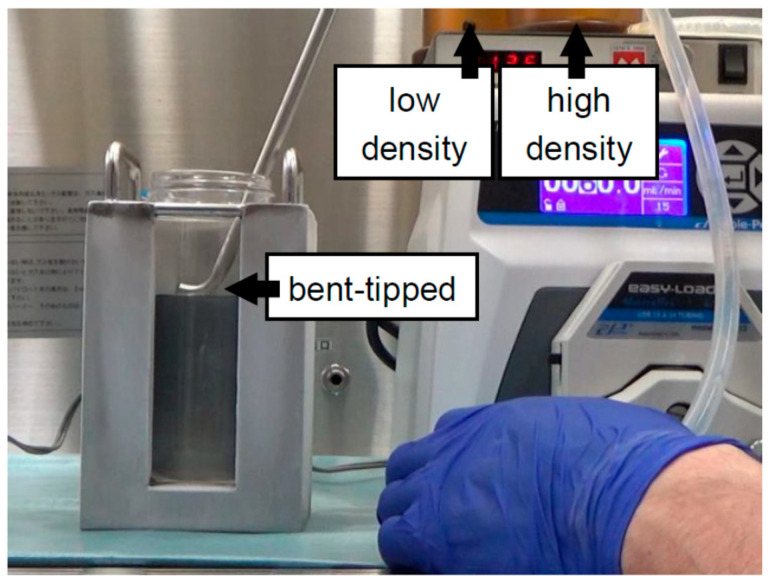
Islet purification using large plastic bottles. A “bent-tipped” stainless pipe was used and loading from a high-density solution to a low-density solution with uploading of the stainless-steel pipe was performed to make a continuous gradient (BP-A method). The BP-A method is similar to COBE purification in that it involves loading from a high-density to a low-density solution. A “bent-tipped” stainless-steel pipe is used to minimize the turbulent flow in the BP-A method.

Before islet purification by continuous density gradient using the COBE 2991 cell processor for clinical islet isolation, researchers used various purification techniques were used including handpicking, serial sieving, and discontinuous density gradient using 50–250 mL tubes/bottles [26,29,48,49]. We have reported that the high shear force involved in the purification method using the COBE 2991 cell processor causes mechanical damage to the islets [50]. The unique shape of the COBE bag with its narrow segment in particular results in strong shear forces [50]. On the other hand, the shear stress can be minimized or eliminated using large cylindrical plastic bottles, because the bottles do not have a narrow segment and no centrifugation is required during tissue loading and the collecting processes of islet purification. Therefore, we evaluated the efficacy of porcine islet purification using 500 mL bottles [18]. The size of the islets purified by the bottle method was significantly larger than that of the islets purified by COBE purification [18]. In the study, we made continuous density gradient in the 500 mL tubes (Figure 2) and loaded less than 20 mL of tissue in one bottle. The method of the bottle purification was similar to that of the COBE purification, and the bottle purification had better efficacy than that of the COBE purification [18]. It has been reported that there is similar efficacy to islet purification in porcine as well as human islet isolation [18,23]. Incidentally, these studies also showed that the top-loading method of digested tissue using bottles was better for the rate of post-purification recovery than the bottom-loading method [18,23].

## 6. Modification of the Bottle Purification Method

**Figure 3:**
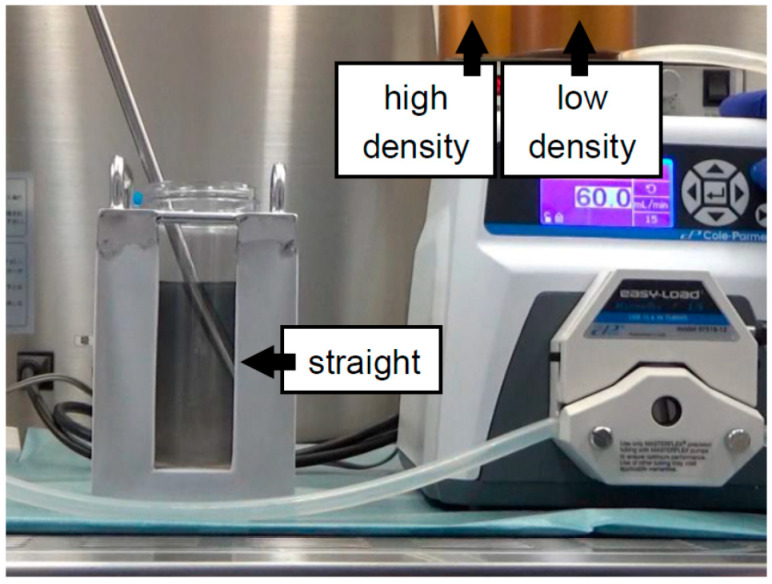
Modifications to create a continuous density gradient for bottle purification. A regular stainless-steel pipe was used and loaded from a low-density solution to a high-density solution; fixing the stainless-steel pipe in place was performed to make a continuous gradient (BP-B method). For the loading of the solutions, the BP-B method was the inverse of the COBE purification and BP-A methods. The technical variability was reduced because the BP-B method fixes the stainless-steel pipe in place.

In our first experiment on bottle purification, a “bent-tipped” stainless-steel pipe was used and loaded from a high-density solution to a low-density solution, with uploading of the stainless-steel pipe being performed to make a continuous gradient (BP-A method) [18] (Figure 2). The BP-A method is similar to COBE purification in that it involves loading from a high-density to a low-density solution. However, there is one weak point to BP-A purification in that there is a turbulent flow during the creation of the continuous density gradient. Although a “bent-tipped” stainless-steel pipe was used to minimize the turbulent flow in the BP-A method, the technician had to upload the pipe carefully to make a continuous density gradient. Therefore, we applied the new method (BP-B method) in the second study [19]. A regular stainless-steel pipe was used and loaded from a low-density solution to a high-density solution, fixing the stainless-steel pipe in place to make a continuous gradient (Figure 3). For loading the solutions, the BP-B method was the inverse of the COBE purification and BP-A methods. The technical variability was reduced because the BP-B method fixes the stainless-steel pipe in place. Although a weak point of the BP-B method is the turbulent flow that removes the pipe after creating a continuous gradient, we verified that the turbulent flow rarely occurs in that action. 

**Figure 4:**
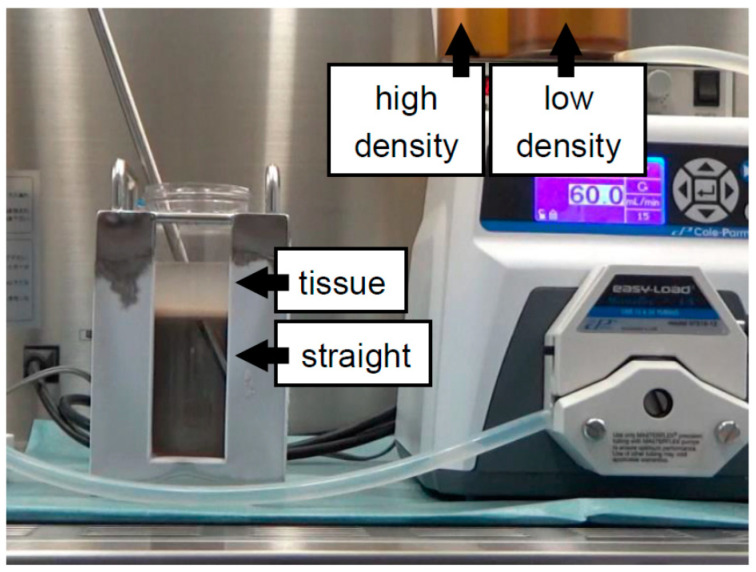
Modification of the timing of tissue loading for bottle purification. Digested tissue is loaded “before” creating a continuous density gradient and then a continuous density gradient is created “under” the tissue using the BP-B technique. The method to load digested tissue “before” creating a continuous density gradient can minimize the technical disparity and time spent loading tissue during islet purification without a reduction in the purification efficacy.

In our third experiment on bottle purification, we evaluated the effect of the timing of tissue loading [22]. In our first and second studies, the digested tissue was loaded “after” creating a continuous density gradient. However, we had to load the digested tissue gently on a continuous density gradient in order to avoid collapsing the gradient when the digested tissue was loaded after creating a continuous density gradient. Therefore, we loaded the digested tissue “before” creating a continuous density gradient in the third study [22] (Figure 4). This method was completely different from the so-called “bottom-loading” method [51], because it involved loading digested tissue and then creating a continuous gradient “under” the tissue using the BP-B technique. The method to load digested tissue “before” creating a continuous density gradient can minimize the technical disparity and time spent loading tissue during islet purification without a reduction in the purification efficacy [22].

**Figure 5:**
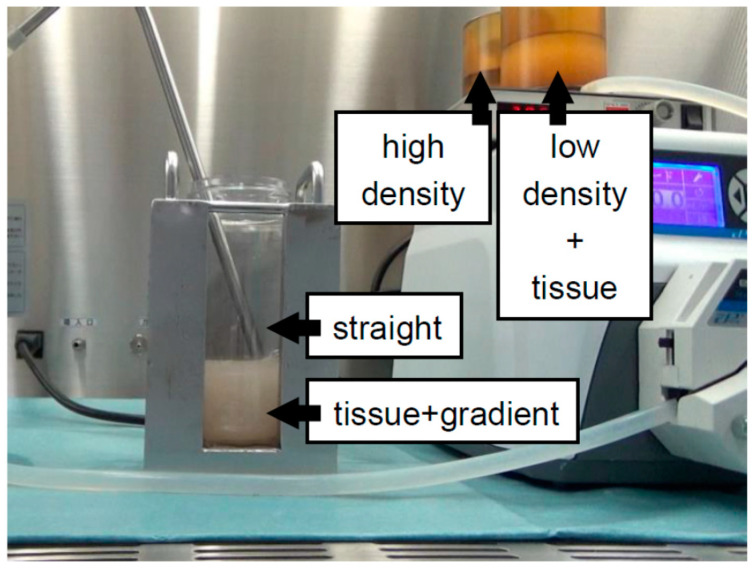
Mixed loading of digested tissue and low-density solution for bottle purification. The method was to mix the digested tissue with a low-density solution and then to make a continuous gradient. The method did not make a pellet of digested tissue beside the wall of bottle, while the purification efficacy was similar to that of the bottle purification with top loading.

In some cases of bottle purification with top loading, a pellet of the digested tissue beside the wall of the bottle was made. Therefore, a fourth study for bottle purification was performed where the method was to mix the digested tissue with a low-density solution and then to make a continuous gradient [21] (Figure 5). The method did not make a pellet of digested tissue beside the wall of the bottle, while the purification efficacy was similar to that of the bottle purification with top loading.

## 7. Future Perspectives

Continuous density gradient with the COBE 2991 cell processor is mainly used for clinical islet purification [1,2,3,4,5,25]. Islet purification techniques have progressed significantly in recent years (Table 1). New gradient media and preincubation solution are developing [7,25,44,52,53] and supplemental/rescue purification has been reported [34,54,55]. It has been reported that the osmolality of the purification solution affects the density of the islets and the exocrine tissue and, therefore, affects purification efficacy. Therefore, some groups including our group have used controlled osmolality solutions for islet purification [11,17,25,56,57,58,59]. Islet purification using large plastic bottles has some advantages over COBE purification [18,19,22,23,24]. The bottle purification method is simple and easy to perform, while the COBE 2991 cell processor is high in cost and is not easy to operate. The bottle purification method could be an alternative method for clinical islet purification.

Another issue for human islet purification is how embedded islets can be separated using current techniques. We frequently observe embedded islets from young donors, while we rarely observe embedded islets from small animals. The density of embedded islets is theoretically between that of islets and that of exocrine tissue because embedded islets consist of islets and exocrine tissue. Therefore, the fraction of embedded islets during islet purification is between that of islets and that of exocrine tissue, commonly called the “middle pure fraction”. Wide construction of middle pure fractions may be important for the separation of embedded islets from exocrine tissue. An iodixanol-controlled density gradient during islet purification [6,25] could be suitable for the separation of embedded islets.

## Figures and Tables

**Table 1:** Islet purification method.

Continuous/ Discontinuous	COBE2991/ Bottle	Solutions	Year	Reference
Discontinuous	50 mL tube	Ficoll	1984	[26]
Discontinuous	250 mL bottle	Ficoll	1988	[26]
Discontinuous	COBE2991	Bovine serum albumin	1989	[29]
Continuous	tube	Bovine serum albumin (Mini-continuous gradient: 11 mL)	1993	[31]
Continuous	COBE2991	Bovine serum albumin	1993	[32]
Continuous	COBE2991	Ficoll	1998	[28]
Continuous	COBE2991	Ficoll	2000	[1]
Continuous	COBE2991	Iodixanol + MK solution	2009	[25]
Continuous	COBE2991	Iodixanol + cold storage/purification stock solution	2011	[6]
Continuous	500 mL bottle	Iodixanol + MK solution (Figure 2)	2012	[18]
Continuous	500 mL bottle	Iodixanol + MK solution (Figure 3)	2016	[19]
Continuous	500 mL bottle	Iodixanol + MK solution (Figure 4)	2018	[22]
Continuous	500 mL bottle	Iodixanol + MK solution (Figure 5)	2018	[21]

## Abbreviations

BSA	bovine serum albumin
IFN-γ	interferon-γ
IL-1β	interleukin-1β
TNF-α	tumor necrosis factor-α
MK	trehalose-containing Kyoto solution with ulinastatin
IK	iodixanol + MK
